# Allosteric Modulators of G Protein-Coupled Dopamine and Serotonin Receptors: A New Class of Atypical Antipsychotics

**DOI:** 10.3390/ph13110388

**Published:** 2020-11-14

**Authors:** Irene Fasciani, Francesco Petragnano, Gabriella Aloisi, Francesco Marampon, Marco Carli, Marco Scarselli, Roberto Maggio, Mario Rossi

**Affiliations:** 1 Department of Biotechnological and Applied Clinical Sciences, University of l’Aquila, 67100 L’Aquila, Italy; irene.fasciani@univaq.it (I.F.); francesco.petragnano@graduate.univaq.it (F.P.); gabriella.aloisi@univaq.it (G.A.); 2 Department of Radiotherapy, “Sapienza” University of Rome, Policlinico Umberto I, 00161 Rome, Italy; francesco.marampon@uniroma1.it; 3 Department of Translational Research and New Technology in Medicine and Surgery, University of Pisa, 56126 Pisa, Italy; carlimarco@outlook.it (M.C.); marco.scarselli@med.unipi.it (M.S.); 4 Institute of Molecular Cell and Systems Biology, University of Glasgow, Glasgow G12 8QQ, UK; mario.rossi@glasgow.ac.uk

**Keywords:** dopamine receptor, serotonin (5-HT) receptor, positive allosteric modulator, negative allosteric modulator, endogenous allosteric modulator, allosteric binding site, orthosteric binding site

## Abstract

Schizophrenia was first described by Emil Krapelin in the 19th century as one of the major mental illnesses causing disability worldwide. Since the introduction of chlorpromazine in 1952, strategies aimed at modifying the activity of dopamine receptors have played a major role for the treatment of schizophrenia. The introduction of atypical antipsychotics with clozapine broadened the range of potential targets for the treatment of this psychiatric disease, as they also modify the activity of the serotoninergic receptors. Interestingly, all marketed drugs for schizophrenia bind to the orthosteric binding pocket of the receptor as competitive antagonists or partial agonists. In recent years, a strong effort to develop allosteric modulators as potential therapeutic agents for schizophrenia was made, mainly for the several advantages in their use. In particular, the allosteric binding sites are topographically distinct from the orthosteric pockets, and thus drugs targeting these sites have a higher degree of receptor subunit specificity. Moreover, “pure” allosteric modulators maintain the temporal and spatial fidelity of native orthosteric ligand. Furthermore, allosteric modulators have a “ceiling effect”, and their modulatory effect is saturated above certain concentrations. In this review, we summarize the progresses made in the identification of allosteric drugs for dopamine and serotonin receptors, which could lead to a new generation of atypical antipsychotics with a better profile, especially in terms of reduced side effects.

## 1. Introduction

Dopamine is a catecholamine neurotransmitter that plays an important role in the central nervous system and exerts its function by binding to dopamine receptors [1]. The major dopaminergic pathways in the brain are the nigrostriatal pathway that originates from the substantia nigra pars compacta and projects to the dorsal striatum, being responsible for the regulation of motor function; the mesolimbic and mesocortical pathways that originate from the ventral tegmental area and project to the limbic system and prefrontal cortex, respectively, and are responsible for motivation, cognition, emotion, memory, and learning; and the tuberoinfundibular pathway that transmits dopamine from the hypothalamus to the pituitary gland and regulates prolactin secretion [2]. While selective degeneration of the nigrostriatal pathway causes Parkinson’s disease, dysregulation of the mesolimbic and mesocortical dopaminergic pathways causes neuropsychiatric disorders, such as schizophrenia, addiction, and attention deficit hyperactivity disorder [3]. While dogma dictates that dopamine dysfunction is central to the pathogenesis of schizophrenia [4], the paradigm-breaking observation was that pure serotonin 5-HT_2A_ antagonist, primavanserin, which does not directly modulate dopaminergic receptor activity, was proven effective in treatment of psychotic symptoms secondary to Parkinson’s disease or dementia [5], suggesting a direct implication of serotonin dysfunction in psychosis.

Since the introduction of chlorpromazine in 1952 [6], dopamine receptors have become the major target for the development of antipsychotic drugs. The existence of dopamine receptors was first revealed in 1972 with the demonstration that dopamine was the major activator of a retinal adenylyl cyclase activity [7], and later on it became apparent that dopamine receptors exist in multiple forms [8]. Five dopamine receptors have been described to date, distinguished by their G-protein coupling, ligand specificity, anatomical distribution, and physiological effects. They are subdivided into two classes: the D_1_-like (D_1_ and D_5_), which are positively coupled to the adenylyl cyclase, and the D_2_-like (D_2_, D_3_, and D_4_), which are negatively coupled to the adenylyl cyclase.

They are plasma membrane proteins composed of seven transmembrane spanning domains connected by three intracellular and three extracellular loops, an extracellular N-terminal domain, and a cytoplasmic C-terminus. The core structure that accepts the dopamine neurotransmitter is buried in a receptor pocket delimited by the transmembrane helices 3, 5, 6, and 7. In particular, dopamine binds to an aspartic acid in the transmembrane region 3 with its protonated amino group, and to serine residues in the transmembrane region 5 with its catechol moiety. This core structure is conserved across the five dopamine receptor subtypes and the other amine receptors, justifying why most of the antipsychotic drugs commercially available targeting the orthosteric site interact with multiple amine receptors.

In recent years, crystallization of dopamine D_2_ [9], D_3_ [10], and D_4_ [11] receptors has become a crucial step to a detailed understanding of the structure and dynamics of receptor binding and functioning and has led the way to the synthesis of more selective and potent drugs, providing refinements to the molecular dynamic models and testable predictions about receptor–ligand interactions [12].

Traditionally, drug discovery programs to target G protein-coupled receptors (GPCRs) have been guided by efforts to develop agonists and antagonists that act at the orthosteric site. However, in recent years, there have been tremendous advances in the discovery of allosteric ligands for GPCRs [13]. Allosteric modulators are compounds that interact with binding sites topographically distinct from the orthosteric site recognized by the receptor’s endogenous agonist and have not evolved to accommodate endogenous ligands [14]. Allosteric modulators show specific advantages, such as the increased selectivity for G protein-coupled subunits and the potential to preserve activity dependence and both spatial and temporal characteristics of endogenous physiological ligands. In addition, recently, a new concept has been introduced that is related to GPCR function, referred to as biased agonism, which entails the capacity of a ligand to preferentially activate either G protein-dependent signaling or G protein-independent signaling [15]. In this review, we summarize the recent discovery of new allosteric compounds for dopamine and serotonin receptors that can influence the underlying molecular mechanisms of schizophrenia. It is beyond the scope of this manuscript to treat allosteric modulators of potential interest for the treatment of schizophrenia that bind to other GPCRs, such as class C metabotropic glutamate receptors; the reader is referred to the following reviews for an in depth description of these subjects [16,17].

## 2. Allosteric Modulation of GPCRs and Influence on Receptor Homo- and Heterodimerization

From the pharmacological standpoint, the simplest classification of the allosteric modulators comprises three types of compounds: positive allosteric modulators (PAMs), which potentiate agonist activity at the orthosteric site; negative allosteric modulators (NAMs), which decrease agonist activity at the orthosteric site; and silent allosteric modulators (SAMs), that bind at allosteric sites and have no effect on the orthosteric site. This latest class does not have an effect per se, but prevent PAM and NAM to bind the allosteric site; as an example, flumazenil is a neutral allosteric modulator of gamma-aminobutyric acid (GABA)_A_ receptor and is used as an antidote to benzodiazepine overdose [18].

**Figure 1:**
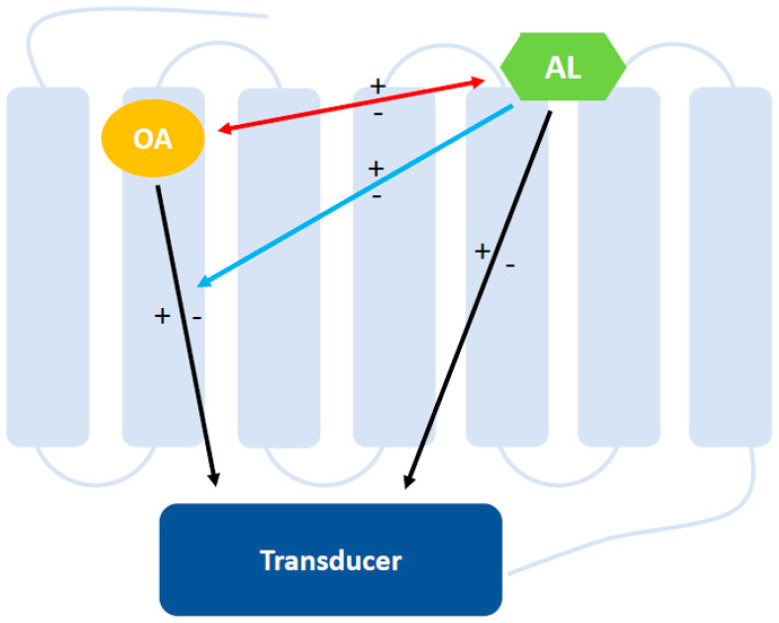
Allosteric modulation of class A G protein-coupled receptors (GPCRs). Orthosteric and allosteric binding sites are topographically distinct in the receptor protein. An allosteric ligand (AL) positively (+) or negatively (-) modulates the orthosteric agonist (OA) affinity (red arrow) and or efficacy (blue arrow) to the receptor. Moreover, allosteric modulators might have a direct agonist (+) or inverse agonist (-) effect on the receptor (black arrow) independent from the orthosteric ligand. The diagram was modified from Conn et al. (2009) [27].

The picture becomes more complicated if we consider that PAMs and NAMs can influence the receptor response to an agonist bound to the orthosteric site affecting either its affinity or its efficacy, as illustrated in Figure 1 [19]. Furthermore, allosteric compounds can directly stimulate or inhibit the receptor with and without modulating the response of an orthosteric agonist, expanding the option of allosteric therapeutic strategies to PAM agonists [20], PAM-antagonists [21], NAM agonists [22,23], and NAM inverse agonists [24,25,26] (Table 1).

Another level of complexity is added by the influence of allosteric ligands on GPCR homo- and heterodimerization. Homo- and heterodimerization have received general recognition as being responsible for tuning, diversifying, and amplifying GPCR signaling, which strongly suggests that very complex interactions take place between ligands and receptor quaternary structures [14,28,29,30]. Agonist or antagonist binding to one protomer of a GPCR homo- or heterodimer can alter the binding and the functional properties of agonist or antagonist interacting with the other protomer, suggesting an allosteric type of interaction between the two protomers [31]. Importantly, the three most abundant dopamine receptor subtypes, D_1_, D_2_, and D_3_, form heteromeric complexes, and D_1_–D_3_, D_1_–D_2_, and D_2_–D_3_ heteromers have different signaling properties compared with the respective monomers in functional assays [32,33,34,35,36]. As we will see later, this phenomenon of allosteric modulation across dimers can be targeted with drugs.

**Figure 2:**
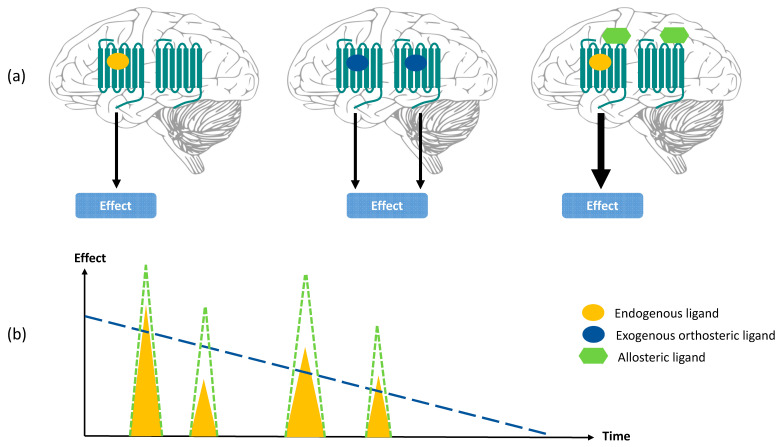
Spatial (**a**) and temporal (**b**) fidelity of positive allosteric modulation of the native orthosteric ligand. (**a**) Endogenous agonist (left panel) is released where it is necessary and activates the receptor in a specific area of the brain. Exogenous orthosteric agonist (middle panel) activates target receptors throughout the brain. Positive allosteric modulator (right panel) is distributed throughout the brain but modifies receptor activity only in the presence of the endogenous ligand, thus maintaining spatial fidelity. (**b**). Endogenous agonist (yellow triangles) is released and cleared quickly, leading to transient signaling effects. Exogenous orthosteric agonist (dashed blue line) occupies receptor persistently, leading to effects that last until the drug is cleared. Positive allosteric modulator (green dashed triangles) achieves physiological activity only when the endogenous agonist is released, thus maintaining temporal fidelity. Image (**b**) was modified from Burford et al. (2015) [39].

**Figure 3:**
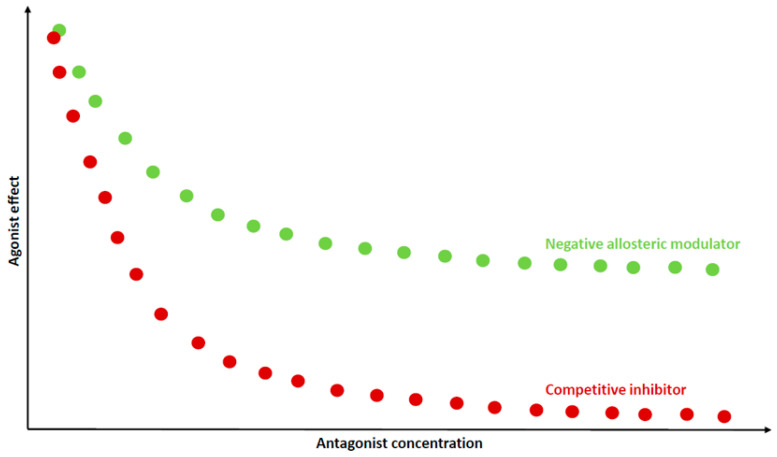
Ceiling effect of negative allosteric modulator. Increasing concentration of the competitive antagonist inhibits the effect of the endogenous agonist until the signaling is quenched (red dots). Increasing concentration of the negative allosteric modulator weakens the signal but does not extinguish it (green dots).

In recent years, great effort has been devoted to the development of allosteric modulators of GPCRs as potential therapeutic agents, mainly because of the several advantages in the use of allosteric modulators with respect to orthosteric ligands [37]:

(a) Receptor selectivity—While orthosteric sites of GPCRs that bind the same or similar endogenous ligands are highly conserved due to evolutionary pressure, allosteric sites are less conserved, allowing for the accommodation of highly selective allosteric modulators. For instance, the negative allosteric modulator SB269652 is highly selective for D_3_ receptor with 100–1000-fold less affinity for nearly all the other amine receptors [38].

(b) Maintenance of temporal and spatial fidelity—Positive allosteric modulators that lack agonistic activity will only exert their effects when the endogenous agonist is present, maintaining its temporal and spatial fidelity. Therefore, in contrast to orthosteric ligands that will indiscriminately activate or inactivate receptors throughout the body, allosteric modulators will have an effect only where the endogenous ligand is released [39] (Figure 2).

(c) Ceiling effect—When the allosteric sites on all available receptors are occupied, no additional changes on the orthosteric sites are possible, and thus the allosteric modulators set a limit to the effect of the orthosteric ligand, i.e., a “ceiling effect” that is dependent on the cooperativity between the two ligands (Figure 3). The main advantage is that allosteric modulators with a defined fold shift of agonist potency may reduce toxicity or avoid overdosing of the patient [39]. This is particularly relevant in schizophrenia, where antipsychotic medication titration to a therapeutic dose is often difficult without unacceptable adverse effects [40].

(d) Probe dependence—The cooperativity of a specific allosteric modulator is probe-dependent [41]; therefore, its allosteric effects depend on the orthosteric ligand under examination and on the receptor signaling analyzed. For instance, the allosteric modulator LY2033298 behaves as a PAM for acetylcholine at the muscarinic M_4_ receptor but as a SAM when tested against the orthosteric antagonist [^3^H]-quinuclidinyl benzilate [42]. This has substantial implication for the classification and the functional characterization of allosteric modulators, which must consider the ligand they modulate and the functional method chosen to assign quantitative parameters [37]. Furthermore, this can have clinical implication when allosteric modulators act on receptors with multiple endogenous agonist ligands. A remarkable example is the glucagon-like peptide 1 (GLP1) receptor, where the small molecule Novo Nordisk compound 2 had no effect on the signaling of the orthosteric peptide agonist GLP1 but significantly potentiated the signaling of oxyntomodulin [43].

(e) Ligand-biased signaling—Traditionally, it has been thought that stimulation of a receptor by ligands activates a defined cascade of signaling pathways. In the last decade, evidence has convincingly demonstrated that ligands can activate multiple pathways downstream from the same receptor. This characteristic of ligands is called signaling bias [44]. While ligand-biased signaling has been extensively reported for many orthosteric ligands, the possibility that allosteric modulators may change the active conformation of a receptor towards one pathway and away from another is still purely theoretic [39]. Nevertheless, “biased allosteric modulation” has been observed for some GPCRs already [17], and could represent a further advantage compared to biased orthosteric signaling by limiting ligand bias only where the endogenous ligand is released. 

(f) Improved chemical tractability—Orthosteric sites often impose stringent limits to the ligand conformation required for binding, while this requirement is less stringent for allosteric sites.

While receptors are physiologically stimulated by endogenous orthosteric agonists, few naturally occurring allosteric modulators have been described [37]. These include the amino acid D-serine for *N*-methyl-l-aspartate (NMDA) receptor [45], the L-phenylalanine and L-tryptophan for calcium receptor [46], the tetrapeptide 5-HT moduline for 5-HT_1B_ receptor [47], and lipoxin A4 for cannabinoid CB_1_ receptors [48]. Furthermore, steroid hormones such as pregnenolone, progesterone, and estradiol exert allosteric effects on cannabinoid, oxytocin, and glycoprotein hormone receptors, respectively [49,50,51]. Finally, ions, cholesterol, and autoantibodies exert allosteric modulation on various receptors (see [52] and references therein). This evidence entails that receptors can be indirectly modulated by changing the concentration of the endogenous allosteric modulator.

## 3. Dopamine and Serotonin Receptors in the Brain and Their Implication in Schizophrenia

The dopamine hypothesis of schizophrenia was first proposed by Van Rossum in 1967 [53]. The origin of this hypothesis dates from early studies involving the use of amphetamine, which worsens psychotic symptoms, such as delusions and hallucinations, and increasing dopamine, while reserpine reduces psychotic symptoms depleting dopamine. This concept has been reinforced by the efficacy of currently-available antipsychotics in controlling these symptoms in most patients with schizophrenia [54]. Conversely, antipsychotic drugs are poorly effective against concomitant cognitive and negative symptoms, such as decreased emotional expression, amotivation, asociality, and anhedonia, which are dependent, at least in part, on the hypo-activity of the mesocortical dopaminergic pathway [55].

Dopamine D_2_-like receptors, the main target of antipsychotic drugs, are highly distributed in different brain regions: dorsal striatum and ventral striatum (the latter including the nucleus accumbens and the olfactory tubercle in rodents), and to a lesser extent substantia nigra, ventral tegmental area, hypothalamus, prefrontal cortex, septum, amygdala, and hippocampus [56]. The less abundant D_3_ and D_4_ receptors are mainly distributed in the limbic regions [57]. This wide distribution accounts for the multiple roles played by these receptors in several physiological conditions. Chronic antipsychotic medication can interfere with most of these conditions, causing extrapyramidal side effects such as parkinsonism, akathisia and tardive dyskinesia, hyperprolactinemia with gynecomastia, and sexual disturbance, and for chlorpromazine and second generation antipsychotics metabolic disorders such as weight gain, hyperglycemia, increased risk of diabetes mellitus, and dyslipidemia [40]. Extrapyramidal side effects are particularly troublesome and are more common with first generation antipsychotics that bind with high affinity to dopamine D_2_ receptors. They appear when more than 80% of the dopamine receptors in the striatum are occupied by the antagonist [58]. Tapering the drug dosage to maintain the receptor occupancy under 80% is often difficult with orthosteric drugs, while it could be more feasible with allosteric drugs, which have a ceiling effect, in terms of keeping the potency of endogenous dopamine above a safe limit.

The main dopaminergic target for antipsychotic drugs is the D_2_ receptor, but many first- and second-generation antipsychotics are also antagonists at D_3_ and D_4_ receptors [59]. Positive symptoms, such as delusions, hallucinations, and thought disorder, are most responsive to anti-dopaminergic drugs, while negative symptoms, such as inappropriate emotion, poverty of speech, and lack of motivation, are resistant to anti-dopaminergic drugs [40].

In this regard, some reports indicate that more balanced D_3_ versus D_2_ dopamine receptor antagonism may be an interesting alternative to treat negative symptoms of schizophrenia [60]. Dopamine D_3_ receptor antagonists influence the electrical activity of dopaminergic neurons in the ventral tegmental area; increase cortical dopamine release; and beneficially affect several cognitive and social features in animal models, such as cognitive flexibility and executive function [61], supporting the concept that D_3_ receptor blockade could optimize the antipsychotic effect of dopaminergic antagonists [58].

Besides being dopamine antagonists, most second-generation antipsychotic drugs are also serotonin receptor antagonists/partial agonists and bind with high affinity to 5-HT_1A_, 5-HT_2A_, 5-HT_2C_, 5-HT_6_, and 5-HT_7_ receptors [59]. Furthermore, serotonin receptors are the target for the action of the hallucinogenic drug lysergic acid diethylamide (LSD) [62]. These observations have stimulated the research on the relation between serotonin receptors and schizophrenia.

5-HT_1A_ receptors are expressed in different areas of the brain, mainly in the prefrontal and occipital cortex, hippocampus, amygdala, and ventral tegmental area. 5-HT_1A_ agonists increase dopamine efflux in the frontal cortex and hippocampus through the inhibition of GABA interneurons and disinhibition of glutamatergic neurons [63]. Many second-generation antipsychotics are 5-HT_1A_ partial agonists and this may be relevant for their mechanism of action. Full 5-HT_1A_ receptor agonists can reduce by antipsychotic-induced catalepsy in animal models. Consequently, the low extrapyramidal side effects of some second-generation antipsychotic drugs could be due to their partial agonism at 5-HT_1A_ receptors [64].

5-HT_2A_ and 5-HT_2C_ receptors are widely distributed in the brain but are most abundant in cortical regions and in limbic structures [65]. Stimulation of 5-HT_2A_ receptors induce dopamine release in cortex in a similar way to of 5-HT_1A_ receptors. Conversely, blockade of 5-HT_2A_ receptors determines a strong reduction in dopamine release in the limbic area that contributes to the favorable profile of atypical antipsychotic drugs. It has been suggested that a strong antagonism at 5-HT_2A_ receptor together with diminished dopamine D_2_ antagonism is the key pharmacological attribute that characterizes second-generation antipsychotics [66]. At variance with 5-HT_2A_ receptors, in general 5-HT_2C_ receptor agonists reduce dopamine release while antagonists have the opposite effect [67]. This suggests that the severity of extrapyramidal side effects may be less severe with second-generation antipsychotics, which have higher affinity for 5-HT_2C_ receptors [68].

As concern 5-HT_6_ and 5-HT_7_, their importance in the mechanism of second-generation antipsychotics is still a matter of debate and requires more studies [59].

## 4. Evidence for Endogenous Allosteric Modulators of Dopamine and Serotonin Receptors

Endogenous allosteric modulators for dopamine and serotonin receptors are listed in Table 2.

Homocysteine, a metabolite of the amino acid methionine, has been proposed as an endogenous allosteric modulator of the dopamine D_2_ receptors by selectively reducing the affinity of D_2_ receptors for dopamine [70]. Mass spectrometric analysis showed that homocysteine forms noncovalent complexes with the two Arg-rich epitopes of the third intracellular loop of the D_2_ receptor [70]. The effect of homocysteine on the specific binding of ^3^H-dopamine in membrane preparations from stably co-transfected chinese hamster ovary (CHO) cells begins at 10 μM concentration. Physiological concentrations of this amino acid range between 5 and 15 μM, and thus the effect on dopamine receptors is minimal or absent. Nevertheless, plasma homocysteine concentrations can rise in particular conditions (hyperhomocysteinemia) to 100 μM or more, thus influencing the dopamine D_2_ receptor activity [101].

As suggested by Agnati et al. (2006) [70] hyperhomocysteinemia could provide a mechanism responsible for the secondary effects of L-3,4-dihydroxyphenylalanine (L-DOPA) treatment in Parkinson’s disease. L-DOPA methylation generates homocysteine by the action of catechol-*O*-methyltransferase (COMT), and hyperhomocysteinemia might contribute to the loss of therapeutic effect of L-DOPA over time due to the negative allosteric properties of homocysteine on D_2_ receptors [102]. Therefore, control of homocysteine levels provides an additional explanation for the positive effects of COMT inhibitors associated with L-DOPA in Parkinson’s disease. A meta-analysis of the homocysteine levels in L-DOPA-treated idiopathic Parkinson’s disease has reported that COMT inhibitors counteract hyperhomocysteinemia [102].

Melanostatin (prolyl-leucyl-glycinamide, PLG) or melanocyte-inhibiting factor-I (MIF-I) is a tripeptide hormone produced in the hypothalamus that inhibits the release of melanocite-stimulating hormone. This small peptide is a positive allosteric modulator for agonists at dopamine D_2_ and D_4_ receptors and has fostered the synthesis of more potent peptide analogues such as 3(*R*)-[(2(*S*)-pyrrolidinylcarbonyl)amino]-2-oxo-1-pyrrolidineacetamide (PAOPA) [69]. Positive allosteric modulators such as MIF-I could potentially be useful in Parkinson’s disease, and in a small number of patients, Barbeau (1975) [103] found its potentiating effect of L-DOPA to be remarkable. Nevertheless, its relevance in this disease remains controversial, as Caraceni et al. (1979) [104] failed to see any behavioral response in patients with Parkinson’s disease under L-DOPA therapy treated with 200 mg intravenous MIF-I. Furthermore, in *N*-methyl-4-phenyl-1,2,3,6-tetrahydropyridine (MPTP)-lesioned marmosets administered concomitantly with different doses of MIF-I and L-DOPA/benserazide, no significant differences were reported in motor function or dyskinesias compared with L-DOPA/benserazide alone, casting serious doubt on the utility of this hormone as an adjunctive therapy in Parkinson’s disease [105]. This certainly does not undermine the potential utility of positive dopamine allosteric modulator in Parkinson’s disease, but further studies are needed to design compounds with clinical effect. One of the limits of this tripeptide could be the unfavorable pharmacokinetic profile, as it could be degraded before reaching the brain or be unable to cross the blood–brain barrier.

Orthosteric binding sites of many GPCRs, including the dopamine D_2_ receptor, are allosterically modulated by ions (Table 2) [106]. The effect of Na^+^ ion on dopamine D_2_ receptor was originally discovered in 1980 by Stefanini et al. [107], showing how sodium enhanced the affinity of certain antagonists up to 10–40 times, most of them substituted benzamide derivatives. The binding pocket of Na^+^ in the dopamine D_2_ receptor is delimited by a cluster of highly conserved polar residues that form a roughly pyramidal region defined by Asp-80 (TMD-II), Ser-121 (TMD-III), Asn-419, and Ser-420 (TMD-VII) at each vertex of the base, and Asn-419 backbone oxygen at the apex [72]. A sodium ion at the center of this site could form nearly ideal interactions, neutralizing the negative charge in this region. As we will see below, Na^+^ can also tune the action of allosteric modulators at the dopamine D_2_ receptor [108].

Another ion that modulates dopamine receptors is zinc. Zinc has a negative effect on the binding of antagonists to both D_1_ and D_2_ receptors [73], and recently it has been shown to have a non-competitive interaction with agonist binding at serotonin 5-HT_1A_ receptors [92]. 

Several endogenous allosteric modulators have also been identified for serotonin receptors, with 5-HT-moduline being the most specific among them. 5-HT-moduline is a tetrapeptide (Leu-Ser-Ala-Leu) that is physiologically released in different regions of the brain, particularly under stress conditions, exerting a negative allosteric effect at the 5-HT_1B_ receptor [91,109]. 

5-HT-moduline specifically interacts with 5-HT_1B_ receptors at nanomolar concentrations, resulting in the desensitization of the receptor. As 5-HT_1B_ auto-receptors have an inhibitory effect on the release of 5-HT, 5-HT-moduline ultimately increases its release [91]. The inactivation of the 5-HT-moduline with intracerebroventricular injections of antibodies in mouse models results in a significant modification of the animal behavior in the open-field and elevated plus maze, well-known laboratory tests for anxiety. Treated mice displayed behaviors consistent with an anxiolytic effect of the antibody, suggesting a potential role of 5-HT-moduline in the control of anxiety [110].

A less selective endogenous allosteric regulator is oleamide, an endocannabinoid-like molecule with sleep-inducing properties, found in cerebrospinal fluid and capable of interacting with and activating cannabinoid CB_1_ receptors [111,112]. Positive allosteric effect of this fatty acid has been reported for serotonin tested on 5-HT_2A_ and 5-HT_2C_ receptors [89,113]. A NAM agonist effect was observed with oleamide on 5-HT_7_ receptors in transfected HeLa cells. Oleamide increased cyclic adenosine monophosphate (cAMP) accumulation if given alone while it reduced the neurotransmitter mediated cAMP accumulation when coapplied with serotonin [89]. A direct negative allosteric modulation at 5-HT_7_ receptors was later shown with radioligand binding assays [90].

Cholesterol is another lipid component that allosterically regulates several GPCRs [52]. As regards serotonin receptors, its depletion both enhances ligand binding function for human 5-HT_1A_ receptors in neuronal cells [88] and reduces serotonin binding and signaling via human 5-HT_7_ receptors [87].

The exact mechanism responsible for lipid allosteric modulation of ligand binding and function of serotonin receptor is not known yet, but both oleamide and cholesterol have a structure expected to perturb the fluidity of membrane lipids. It is therefore possible that these compounds operate by perturbing lipid matrices either free or in complex to proteins and protein assemblies [114].

## 5. Exogenous Allosteric Modulator of Dopamine Receptors

The field of exogenous allosteric modulators of GPCRs has grown exponentially in the last 20 years. The advantages that these compounds could bring to the clinic have fostered their search, with four of them eventually being approved for clinical use: the positive allosteric modulator of the calcium-sensing receptor cinacalcet used for primary and secondary hyperparathyroidism and severe hypercalcemia; the allosteric antagonist of purinergic P2Y_12_ receptor ticagrelor as an anti-thrombotic; the negative allosteric modulator maraviroc for the C-C chemokine CCR_5_ receptor; and plerixafor for the C-X-C chemokine CXCR_4_ receptor, used for HIV infection and bone marrow transplantation. Many other molecules are currently in clinical trial [115]. This tremendous progress was made possible by GPCR crystal structures, a variety of functional assays, and in silico computer-based modeling that have improved and optimized the design and development of these drugs.

Early evidence of allosteric modulation for dopamine receptors came with the work of Hoare et al. (2000) [85] on the potassium-sparing diuretic drug amiloride and its analogues. These compounds accelerate the dissociation of the radioligand [^3^H]-SCH-23390 from D_1_ and [^3^H]-spiperone from D_2_ and D_3_ receptors. In equilibrium binding (pseudo-competition) experiments, data generated with amiloride and its analogues best fitted a model where the modulators bind both to the primary and allosteric sites of the receptor. Furthermore, these compounds reduced dopamine potency in [^35^S]-guanosine 5’-*O*-(γ-thio]triphosphate) (GTPγS) binding at the D_2_ receptor. Amiloride and its congeners are not specific for dopamine receptors, but many other class A GPCRs are affected by these compounds, suggesting the presence of a highly conserved amiloride binding site. Amiloride, as a diuretic drug, acts through the blockade of renal epithelial sodium channels, and an intriguing hypothesis states that it could bind to the sodium ion site of GPCRs [116]. The negatively charged carboxylate of sodium ion site Asp residue in transmembrane region II of class A GPCRs (Asp-80 in D_2_ receptor) supposedly interacts with the positively charged guanidinium group present in all amilorides [116]. Likely, the study of amilorides will advance our comprehension of the mechanism of action of Na^+^ ion in GPCR function. However, it is not clear whether it will consent the design of highly selective receptor compounds.

SCH-202676 is another compound that has attracted early attention as an allosteric modulator of several structurally distinct GPCRs, including the dopamine D_1_ and D_2_ receptors [117]. Afterwards, it was discovered that this compound alters GPCR function via modification of sulfhydryl groups rather than an authentic allosteric mechanism [118].

The first specific dopamine receptor allosteric modulator discovered was PAOPA [69]. As mentioned above, PAOPA is a peptide deriving from the conformational constrain of the endogenous allosteric modulator melanostatin. It is much more potent than melanostatin and displays positive modulation of agonist binding to the D_2_ and D_4_ receptors, whereas agonist binding to the D_1_ and D_3_ receptors remains unaffected [69]. The effect of this compound has been tested in vivo on the agonist-induced rotational behavior in 6-hydroxydopamine (6-OHDA)-lesioned rats [119]. In this model, the unilateral dopamine denervation induces the super-sensitivity of dopamine receptors in the lesioned site, which in turn causes a contralateral rotation of the animal upon agonist stimulation. In this well-known model of Parkinson’s disease, PAOPA was found to potentiate the contralateral rotation induced by either apomorphine or L-DOPA with a potency 100-fold higher than its endogenous counterpart melanocortin [119].

Furthermore, in a model of social withdrawal caused by sub-chronic treatment with the non-competitive NMDA receptor antagonist MK-801, PAOPA increased the amount of time spent in social interaction in comparison to control animals [120]. These results suggest that positive allosteric modulators could be helpful for the treatment of social withdrawal and related negative symptoms associated with schizophrenia. In contrast to positive symptoms such as delusions and hallucinations, cognitive and negative symptoms in schizophrenia are dependent, at least in part, upon dopamine depletion in the prefrontal cortex [55]. PAOPA also attenuated haloperidol-induced vacuous chewing movements in rat, a model of human tardive dyskinesia, a troublesome collateral effect that occurs after years of treatment with antipsychotics [121]. Other positive allosteric modulators of D_2_ and D_3_ receptors have been developed in the last ten years and are reported in Table 2.

A positive allosteric modulator, LY3154207, was also developed for the dopamine D_1_ receptor by Hao et al. (2019) [77]. While this compound is not suitable for clinical use due to its scarce solubility, a novel cocrystal form with superior solubility was discovered and advanced to clinical development as a potential first-in-class D_1_ positive allosteric modulator and is now in phase 2 studies for Lewy body dementia. Dopamine D_1_ receptors have a special role in maintaining higher cognitive functions. In particular, working memory, attention, and executive functions are improved by an optimal prefrontal D_1_ receptor activation [122]. Furthermore, it should be emphasized that atypical antipsychotics such as clozapine, increase dopamine release, which will increase D_1_ tone [123]. This action could contribute to the therapeutic efficacy of D_2_ antagonists by improving higher cognitive functions. While orthosteric D_1_ agonists have the disadvantage of having a narrow therapeutic window, positive allosteric modulators could fulfill the task to give an optimal prefrontal D_1_ receptor activation. In accordance with this conjecture, a dopamine D_1_ receptor-positive allosteric modulator, namely, dichlorophenylacetyl 5-(1-hydroxy-1-methyl-ethyl) tetrahydroisoquinoline (DETQ), demonstrated the potential efficacy of D_1_ potentiators for the treatment of cognitive deficits and negative symptoms in schizophrenia [124].

The first negative allosteric modulator of dopamine receptors to be identified was the small molecule SB269652 [14,79]. This compound has been extensively characterized and is treated together with the other negative allosteric modulators in the following paragraph.

## 6. SB269652—a Prototypical Negative Allosteric Modulator of Dopamine D_2_ and D_3_ Receptors

SB269652 was originally synthesized by SmithKline Beecham in the effort to find new selective dopamine D_3_ receptor antagonists [125,126]. Therefore, the radiolabeled [^3^H]SB269652 shows a high binding affinity and selectivity for the dopamine D_3_ receptor [38].

**Figure 4:**
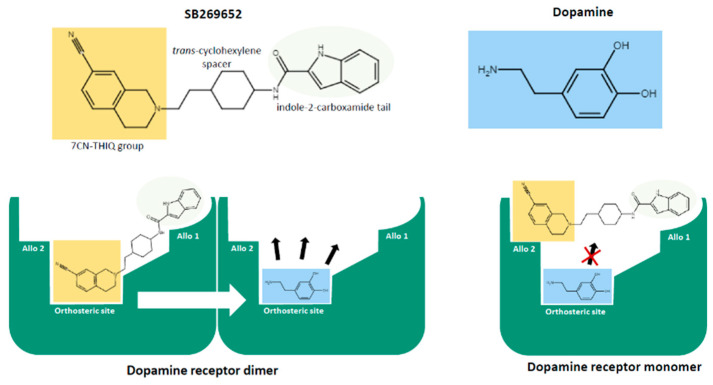
Hypothetical binding mode of SB269652 to dopamine receptor dimer and monomer. SB269652 is composed of three chemical parts, the 7CN-THIQ group, the *trans*-cyclohexylene spacer in the middle, and the indole-2-carboxamide tail. On the left, SB269652 binds in a bitopic mode to one protomer of the dopamine dimer, the 7CN-THIQ group to the orthosteric site and the indole-2-carboxamide group to the allosteric site (Allo1), to influence dopamine binding on the second protomer (black arrows) through an effect across the dimer (white arrow). On the right, SB269652 binds to the dopamine-occupied receptor and prevents agonist dissociation from the monomer (crossed black arrow). In this last configuration, the 7CN-THIQ group could engage an additional allosteric binding site (Allo2). Diagram was modified from Rossi et al. (2017) [14].

In 2010, Silvano et al. [79] discovered that this compound is an atypical, negative, allosteric modulator for D_2_ and D_3_ receptors, having both an orthosteric and an allosteric nature. The orthosteric/allosteric nature for D_2_ and D_3_ receptors was confirmed in radioligand binding and in functional experiments. Later on, Lane et al. (2014) [127] explained the reason for this unusual behavior. They showed that SB269652 is a bitopic compound that is composed of two pharmacophores bridged by a spacer and is characterized by the ability to simultaneously bind to the orthosteric and allosteric sites of the dopamine receptor. The bitopic nature of SB269652 was revealed by fragmentation of the molecule in the two pharmacophores: the 7-cyano-tetrahydroisoquinoline (7CN-THIQ) moiety and the indole-2-carboxamide fragment. In contrast to SB269652, the 7CN-THIQ fragment was able to inhibit dopamine action, in a competitive manner, whereas the other active component, the indole-2-carboxamide fragment, inhibited dopamine action in a noncompetitive manner [127]. The bitopic nature of SB269652 does not explain per se the allosteric action of this compound, but Lane et al. (2014) [127] provided evidence that the allosteric action of SB269652 occurs across dopamine receptor dimers [128]. To summarize, SB269652 binds in a bitopic mode to one protomer of a dopamine D_2_ or D_3_ receptor dimer, the 7CN-THIQ to the orthosteric site and the indole-2-carboxamide to the allosteric site. Then, it exerts an allosteric effect on the binding of the orthosteric ligand to the other protomer of the dimer. In other words, SB269652 behaves as a competitive antagonist at receptor monomer and as a negative allosteric modulator across receptor dimer (bottom left panel of Figure 4).

This “binding” mode would in principle explain the atypical behavior of SB269652 in binding and functional experiments. Nevertheless, some evidence challenges this interpretation and suggests that additional complexity underlies the function of this compound. The affinity of a compound for its target is related to its association and dissociation rate constants and is quantified by K_D_ = K_off_/K_on_, where K_D_ represents the equilibrium dissociation constant and K_off_ and K_on_ represent the dissociation and association rate constants, respectively [27,129]. Consistent with this notion, when an allosteric compound modifies the affinity of a ligand for the orthosteric site, its dissociation and association rate constants must change accordingly. Usually, negative allosteric compounds increase the dissociation rate constant and/or decrease the association rate constant of a ligand at the orthosteric site. Strikingly, Silvano et al. (2010) [79] demonstrated that SB269652 does not behave as a common negative allosteric modulator; in fact, it largely reduces both radioligand association and dissociation rate constants for D_2_ and D_3_ receptors. Methoctramine, another bitopic ligand, behaves in the same way at muscarinic M_2_ receptors [130]. It competes with orthosteric ligands by simultaneously binding to both the orthosteric and the allosteric binding sites of M_2_ receptor. In addition, methoctramine binds with a low affinity to *N*-methylscopolamine-occupied receptors by interacting solely with the allosteric binding site of M_2_. In dissociation binding experiments with *N*-[^3^H]-methylscopolamine-prelabeled M_2_ muscarinic receptors, methoctramine inhibits radioligand dissociation, and it was proposed that the radioligand was trapped in the orthosteric site of M_2_ by methoctramine [130]. In a similar manner, SB269652 could bind to the radioligand-occupied receptor exclusively at the allosteric site and prevent ligand dissociation. Thus, SB269652 could have different binding pose depending on whether the receptor is occupied by the radioligand or not. The 7CN-THIQ head group could be oriented to bind different regions of the dopamine receptor according to the receptor occupancy status (bottom right panel of Figure 4). Indeed, a flip-flop mechanism of binding for bitopic ligands was previously suggested by Lane et al. (2013) [131].

SB269652 has become a leading compound for the design and synthesis of more potent allosteric modulators. For instance, Shonberg et al. (2015) [84] synthesized a series of compounds that bind to dopamine D_2_ receptors in a bitopic manner but with higher affinity compared to SB269652. They modified the three essential chemical parts of SB269652: the 7CN-THIQ, the indole-2-carboxamide moieties, and the *trans*-cyclohexylene linker. Substitution of the 7CN-THIQ head group with chemical structures that are privileged scaffolds for dopamine D_2_ receptors resulted in compounds with the highest increase in affinity but poor allosteric effect. Conversely, substitution of the 7CN-THIQ with chemical structures without scaffold structure maintained the allosteric characteristics in an unaltered way [84]. Furthermore, they showed that the length and the chemical characteristics of the *trans*-cyclohexylene linker is important for the correct orientation and binding of the 7CN-THIQ and the indole-2-carboxamide groups to the orthosteric and allosteric sites, respectively. 

The effect of modifications in the indole-2-carboxamide moiety of SB269652 was also analyzed. Data from D_2_ and D_3_ chimeric receptors showed that the SB269652 allosteric binding site on D_2_ and D_3_ receptors is delimited by extracellular loops I and II [79]. This concept was strengthened by Lane et al. (2014) [127], who demonstrated that the cyclohexyl and indolic NH residues of SB269652 bind, respectively, to valine 91 and glutamic acid 95, located at the extracellular end of transmembrane region II of the D_2_ dopamine receptor. Furthermore, Verma et al. (2018) [132] showed that hydrogen binding of the indole-2-carboxamide moiety to glutamic acid residues of D_2_ and D_3_ receptors is dynamic and is formed intermittently.

In order to design analogs with an improved allosteric effect, SB269652 was broken down to its indole-2-carboxamide group (compound 3 in Mistry et al. (2015) [83]). The 1H-indole-2-carboxamide moiety behaved as a pure allosteric drug at D_2_ receptors, even though its affinity and negative cooperativity were weaker than SB269652. This molecule had similar affinity and negative allosteric effects for D_3_ dopamine receptors compared to the D_2_ receptors, which strongly suggested that the difference in affinity between D_2_ and D_3_ receptors depends on the binding of the 7CN-THIQ moiety to the orthosteric site of the two receptors [38,79]. Furthermore, Mistry et al. (2015) [83] confirmed that the residues valine 91 and glutamic acid 95 of D_2_ receptor were crucial for maintaining the allosteric properties of the 1H-indole-2-carboxamide derivative. Interestingly, Mistry et al. (2015) [83] also found that the 1*H*-indole-2-carboxamide moiety in extracellular signal-regulated kinase (ERK)1/2 functional assays reduced either dopamine potency and efficacy, while in dopamine-mediated GTP*γ*S recruitment and cAMP production, it reduced only dopamine potency, indicating a biased effect of this compound on receptor function. 

When analogs of the 1*H*-indole-2-carboxamide with N-substitutions in the carboxamide group were generated, the linear increase in size of the alkyl substituents (from *N*-ethyl to *N*-butyl) improved both affinity and negative cooperativity. The N-butyl substituent (compound 11d in Mistry et al. (2015) [83]) was the compound with the most pronounced allosteric effect, which suggests that alkyl substituents bind to a hydrophobic part of the receptor core. Furthermore, analogues in which the NH group of the indole ring could not form hydrogen bonds did not show any allosteric property, further supporting the concept that the NH group of the indole ring binds to the glutamic acid 95 of the D_2_ receptor [83]. Recently, Draper-Joyce et al. (2018) [108] showed that the presence of a Na^+^ within the conserved Na^+^-binding pocket of dopamine D_2_ receptor is required for the negative allosteric effect of SB269652; thus, SB269652 acts synergistically with Na^+^ to modulate the binding of orthosteric ligands. 

The 1H-indole-2-carboxamide moiety optimized with the N-butyl substituent was anchored to the 7CN-THIQ group in order to reconstruct a bitopic molecule; the resulting molecule (compound 36 in Mistry et al. (2015) [83]) showed an increase in affinity (81 nM) for the D_2_ receptor and a similar negative allosteric cooperativity compared with its leading compound SB269652. It is worth noting that compound 18d from the study of Shonberg et al. (2015) [84], with an hexyl linker in place of the butyl linker, showed higher affinity for the D_2_ receptor and similar negative allosteric cooperativity as compared to compound 36 of Mistry et al. (2015) [83]. 

One important consequence of the work of Mistry et al. (2015) [83] is that the bitopic pose of SB269652 is not essential for its allosteric effect as compound 11d retains the allosteric properties. Furthermore, it casts further doubt on the allosteric mechanism across the dimer proposed above [127].

During the last few years, optimization of the structure of SB269652 led to more potent allosteric modulators for D_2_ and D_3_ receptors. An interesting approach was used by Kumar et al. (2017) [78], who synthesized a series of molecules where the primary and secondary pharmacophores were derived from SB269652 and SB277011A (a competitive antagonist), whose structural similarity and pharmacological disparity provided the perfect templates for SAR investigation. Incorporating a trans-cyclopropylmethyl linker between the 7CN-THIQ and the 1H-indole-2-carboxamide moieties and manipulating linker length, the researchers identified two bivalent noncompetitive D_3_-selective antagonists, 18a and 25a [78]. These compounds further delineate SAR associated with allosterism at D_3_ receptor and provide leads toward novel drug development. Furthermore, relatively subtle changes to structure and orientation of the secondary pharmacophore can cause a switch between apparently allosteric and competitive antagonism. More recently, Kopinathan et al. (2019) [80] designed a compound (11g) with sub-nanomolar affinity for the D_2_ receptor (K_B_ = 0.148 nM), which showed a 100-time increase in cooperativity for dopamine and acted to decrease the potency and maximal effect of dopamine. Other negative allosteric modulators for dopamine receptors can be found in Table 2. 

## 7. Exogenous Allosteric Modulator of Serotonin Receptors

In contrast with the exponential growth of scientific literature regarding the allosteric modulators of dopamine receptors, publications on allosteric modulators of serotonin receptors are still limited, except for the 5-HT_2C_ (Table 2) and 5-HT_3_ receptors. Allosteric compounds for the 5-HT_3_ receptor will not be treated here, as this receptor does not belong to the GPCR family but to ion channels [133,134]. 

Starting with the 5-HT_1A_ receptor, the synthetic cannabinoid AM2201 was described as a positive allosteric modulator [93]. This compound was examined for its effect on 5-HT_1A_ agonist-activated G protein-coupled inwardly rectifying potassium channel currents in neurons in vitro and on the hypothermic response to 5-HT_1A_ receptor stimulation in vivo. AM2201 potentiated both 5-HT_1A_ effects, suggesting a positive allosteric modulation at this receptor. Further evidence will be needed to delineate the allosteric nature of this compound, especially in cell lines expressing recombinant 5-HT_1A_ receptors.

Ergotamine is an ergot-derived alkaloid and is used for the treatment of acute migraine attacks for its effect on 5-HT_1B_, 5-HT_1D_, and 5-HT_1F_ receptors. The crystal structure of the ergotamine bound 5-HT_1B_ receptor was resolved in 2013 [135,136] and showed the ligand binding to the orthosteric pocket and an additional binding pocket close to the extracellular loops. Interestingly, by over imposing the crystal structure of the ergotamine bound to the 5-HT_1B_ receptor with the muscarinic M_2_ receptor bound with the positive allosteric modulator LY2119620 [137], it becomes apparent that LY2119620 occupies the same extracellular region as the tripeptide portion of ergotamine. These similar poses in both the M_2_ and 5-HT_1B_ receptors suggest that the location of the extracellular allosteric site for class A GPCRs is quite similar. Moreover, ergotamine likely functions as a bitopic ligand and occupies both the orthosteric and the putative extracellular allosteric site in the serotonin 5-HT_1B_ receptor, opening to the potential design of novel allosteric modulators for serotonin receptors [138].

As regards the 5-HT_2A_ receptors, only a weak positive allosteric effect on the function of serotonin at this receptor has been reported for (*S*)-glaucine, a tetrahydroisoquinoline alkaloid [94]. Nevertheless, an indirect allosteric effect has been described in cells co-expressing the 5-HT_2A_ and the metabotropic glutamate mGlu_2_ receptor [139,140,141]. In particular, as shown by studies in vitro and in vivo, the 5-HT_2A_-mGlu_2_ complex enhances the activity of the 5-HT_2A_ component towards Gα_i_, and less towards Gα_q_, and the activation of the mGlu_2_ component of the complex arrests the hallucinogenic properties induced by 5-HT_2A_ receptor agonists, such as LSD. Mechanistically, the mGlu_2_ monomer has an allosteric negative effect on 5-HT_2A_-mediated Gα_q/11_ activation, while enhancing its Gα_i/o_ activity. As we already mentioned above for SB269652, this phenomenon could be regarded as an allosteric modulation across heterodimers (Figure 4). As we will see in the next paragraph this has strong implication for the discovery of new treatments for schizophrenia.

A piperazine-linked phenyl cyclopropyl methanone was found by Singh et al. (2019) to be a positive allosteric modulator of 5-HT_2C_ and negative allosteric modulator of 5-HT_2B_ receptors [95]. This compound (58 in Singh et al. (2019) [95]), as a positive allosteric modulator of 5-HT_2C_, increases the E_max_ of 5-HT to 139%, and as a negative allosteric modulator of 5-HT_2B_, decreases the EC_50_ of 5-HT 10 times without affecting the E_max_. Molecular docking studies revealed that this allosteric compound binds to the predicted allosteric site on 5-HT_2B_ [135,136] and 5-HT_2C_ receptors [142].

Among the 5-HT_2_ receptor family, the 5-HT_2B_ has received much less attention with regard to its functional role within the central nervous system. Nevertheless, accumulating evidence in the last decade highlights its key role on dopamine neuron activity [143]. Selective blockade of 5-HT_2B_ receptors decreases the firing activity of dopamine neurons in the ventral tegmental area and, most remarkably, those projecting to the shell of the nucleus accumbens, involved in the cognitive processing of reward [144]. Opposite changes were observed in median prefrontal cortex, where the selective 5-HT_2B_ receptor antagonists RS127445 and LY 266097 were able to increase dopamine outflow [145]. These conflicting results have been attributed to a functional interaction with 5-HT_1A_ receptors, triggered by a cortical serotonin outflow and involving polysynaptic cortical–subcortical pathways [143]. This unique pattern of effects on the dopaminergic system supports 5-HT_2B_-negative allosteric modulators as possible therapeutic strategies for dopamine-related neuropsychiatric diseases, especially in disorders requiring an independent modulation of the activity of ascending dopamine projections such as in schizophrenia [143].

In contrast to the other 5-HT receptor subtypes, much more work has been done to find allosteric modulator of the 5-HT_2C_ receptor [98]. In 2003, chemical library screening resulted in the discovery of an analogue of the antibiotic lincomycin, PNU-69176E, as a selective positive allosteric modulator of 5-HT_2C_ receptor [146]. The optimization of this compound led Ding et al. (2012) [147] to generate PNU-69176E and Wild et al. (2019) [100] to design, synthesize, and characterize a series of new chemical entities as selective 5-HT_2C_-positive allosteric modulators. Among them, CYD-1-79 potentiated 5-HT-evoked intracellular calcium release in cells stably expressing the human 5-HT_2C_ but not the closely related 5-HT_2A_ receptor. Furthermore, it exhibited a favorable pharmacokinetic profile, potentiated 5-HT_2C_-mediated suppression of spontaneous locomotor activity in rodent, and attenuated impulsive action and sensitivity to cocaine-associated cues in a preclinical self-administration model [98]. Finally, in a last optimization hit, the same group synthetized and tested a new positive allosteric modulator of 5-HT_2C_ receptor, CTW0415 [97]. On the basis of the 4-alkylpiperidine-2-carboxamide scaffold, they optimized the undecyl moiety at the 4-position with phenyl-containing fragments to reduce rotatable bonds and lipophilicity. This compound modulated the maximal effect of 5-HT-induced intracellular Ca^2+^ release in 5-HT_2C_ receptor transfected cells but not the potency of the neurotransmitter. Furthermore, it showed high affinity and selectivity for the 5-HT_2C_ receptor allosteric site as its activity started at concentration as low as 1 nM. Another positive allosteric modulator for the 5-HT_2C_ receptor, VA012, has been proposed for the treatment of obesity by García-Cárceles et al. (2017) [96]. This compound exhibited dose-dependent enhancement of serotonin efficacy at 5-HT_2C_ receptors, with no significant off-target activities, and low binding competition with serotonin or other orthosteric ligands. VA012 was very active in feeding inhibition in rodents, an effect that was not related to the activation of 5-HT_2A_ receptors. Furthermore, the combination of VA012 with sertraline, a selective serotonin reuptake inhibitor that increases the level of 5-HT in the synaptic cleft, potentiated the anorectic effect. This series of allosteric modulators of 5-HT receptors is paving the way for the discovery of novel therapeutic drugs. 

## 8. Allosteric Modulators as a New Class of Antipsychotics

Dopamine and serotonin receptors remain the main targets for the development of antipsychotic drugs. The use of these drugs, mainly atypical antipsychotics, has increased over time, not only in schizophrenia and bipolar disorder, but also for treatment of dementia in elderly populations, posttraumatic stress, and obsessive–compulsive disorders [148,149,150]. Available antipsychotics are mainly dopamine antagonists at the orthosteric binding site; one of the main limits of these drugs is the risk of high plasma concentration, with a level of D_2_ receptor occupancy higher than the threshold for extrapyramidal adverse reactions (about 80%). Conversely, as we mentioned above, negative allosteric modulators of dopamine receptors have a potential ceiling effect (Figure 3)—they could down-tune dopamine signaling and eliminate delusions and hallucinations but limit the risk of adverse reactions for the ceiling effect. Furthermore, the high similarity of D_2_ and D_3_ receptors allows for the design of allosteric drugs that have negative allosteric effects on both receptors [14]. Since inhibition of dopamine D_3_ receptor increases cortical dopamine release, beneficially influencing cognitive flexibility and executive function [61], drugs with well-balanced negative dopamine cooperativity at both D_2_ and D_3_ receptors could have effect on both positive and negative symptoms of schizophrenia. Moreover, treatment-resistant cognitive deficits could be improved by the increase of D_1_ tone in prefrontal cortex [123]. In fact, in contrast with orthosteric D_1_ agonists, allosteric D_1_ agonists maintain spatial and temporal selectivity. This property can be exploited to have safer drugs to use as an adjunctive therapy for schizophrenic patients who have cognitive-resistant deficits. As a matter of fact, the novel D_1_ receptor-positive allosteric modulator LY3154207 has now reached the clinic in phase 2 studies for Lewy body dementia [77]; it would be interesting in future to investigate whether it also improves cognitive symptoms in schizophrenia.

On this topic, pimavanserin is a new atypical antipsychotic drug approved for the treatment of psychosis in Parkinson’s disease, and is pending approval for the treatment of schizophrenia, Alzheimer’s disease, and major depressive disorder. Unlike other antipsychotics, pimavanserin is not a dopamine receptor antagonist but acts as an inverse agonist with nanomolar affinity at 5-HT_2A_ and has 40 times lower affinity for 5-HT_2C_ receptors [151]. This drug breaks the taboo that antipsychotics must have an effect on dopaminergic receptors. Allosteric modulators of 5-HT_2A_ receptors are still to come, but 5-HT_2A_-mGlu_2_ heterodimer complexes in the somatosensory cortex might already represent an important pharmacological target in schizophrenia [139]. For instance, Fribourg et al. (2011) [152] demonstrated that 5-HT_2A_-mGlu_2_ heterodimerization was crucial to determine the coupling to Gα_i/o_ or Gα_q/11_ proteins, and different drugs may favor the coupling to either signaling pathways. Interestingly, in schizophrenia, mGlu_2_ downregulation and 5-HT_2A_ upregulation may be associated with an increase of Gα_q_ coupling at the expense of Gα_i_ signaling. Moreover, atypical antipsychotics such as risperidone and clozapine invert the 5-HT_2A_-mGlu_2_ heteromer activity in favor of Gα_i_ coupling, restoring the normal physiological conditions [59]. In concordance with this view, the mGlu_2_-positive allosteric modulator SAR18645 improves memory and attention deficits in translational models of cognitive symptoms associated with schizophrenia [153]. These results further confirm the relevance of the 5-HT_2A_-mGlu_2_ receptor complex in regulating the sensory functions in the somatosensory cortex, which may be disrupted in schizophrenia, offering a unique opportunity to allosterically modulate a receptor through another receptor only in specific areas of the brain where heterodimers are formed.

As we illustrated in this review, many allosteric modulators of both dopamine and serotonin receptors have been discovered thus far and it is likely that in the next years some of these compounds will enter clinical trials. Just as it happened 50 years ago for chlordiazepoxide in the treatment of anxiety and sleep disturbance [154], allosteric drugs for dopamine and serotonin receptors could improve the treatment of schizophrenia, potentiating the therapeutic benefits and reducing adverse reactions.

## Figures and Tables

**Table 1:** Classification of the various allosteric modulators and description of their effect.

Allosteric Modulator	Description of the Effect
PAM (positive allosteric modulator)	Increases orthosteric ligand affinity and/or efficacy
NAM (negative allosteric modulator)	Decreases orthosteric ligand affinity and/or efficacy
SAM (silent allosteric modulator)	Does not affect orthosteric ligand activity, but prevents other modulators from binding to the allosteric site, thus inhibiting their modulation
PAM agonist	Works like PAM, but also functions as an agonist with and without the orthosteric ligand it modulates
PAM antagonist	Works like PAM, but also functions as an antagonist and lowers the efficacy of the orthosteric ligand
NAM agonist	Works like NAM, but also as an agonist with and without the orthosteric ligand it modulates
NAM inverse agonist	Works like NAM, but also functions as an inverse agonist without the orthosteric ligand it modulates

**Table 2:** Allosteric modulators of dopamine and serotonin GPCRs.

**Allosteric Modulators of Dopamine Receptors**			
**Endogenous Molecule**	**Effect**	**Target**	**Reference**
Melanostatin or PLG	PAM	D_2_	[69]
Homocysteine	NAM	D_2_	[70]
H^+^	NAM	D_2_	[71]
Na^+^	NAM	D_2_	[72]
Zn^2+^	NAM	D_1_–D_2_	[73]
**Exogenous Molecule**	**Effect**	**Target**	**Reference**
DETQ	PAM	D_1_	[74]
[5-Fluoro-4-(hydroxymethyl)-2-methoxyphenyl](4-fluoro-1hindol-1-yl)methanone	PAM	D_2_–D_3_	[75]
MLS6585 and MLS1082	PAM	D_1_	[76]
LY3154207	PAM agonist	D_1_	[77]
PAOPA	PAM	D_2_	[69]
Compounds 18a and 25a	NAM	D_3_	[78]
SB269652	NAM	D_2_–D_3_	[79]
Compounds 2 and 11g	NAM	D_2_	[80]
Compounds 9d, 9i and 9h	NAM	D_2_	[81]
Compounds 14a, 14i, 17, 19, 39a, 43a, 47a,b	NAM	D_2_	[82]
Compounds 11d and 36	NAM	D_2_	[83]
Compounds 18a, 18b, 18d and 25f	NAM	D_2_	[84]
Amiloride and amiloride analogues	NAM	D_1_–D_2_–D_3_–D_4_	[85]
Indoloquinolizidine-peptide 28	NAM agonist	D_1_	[86]
**Allosteric Modulators of Serotonin Receptors**			
**Endogenous Molecule**	**Effect**	**Target**	**Reference**
Cholesterol	PAM	5-HT_1A_–5-HT_7_	[87,88]
Oleamide	PAM agonist NAM agonist	5-HT_1A_–5-HT_2A_ 5-HT_2C_–5-HT_7_	[89,90]
5-HT moduline	NAM	5-HT_1B_	[91]
Zn^++^	NAM	5-HT_1A_ –5-HT_7_	[92]
**Exogenous Molecule**	**Effect**	**Target**	**Reference**
AM2201 and JWH-018	PAM	5-HT_1A_	[93]
(S)-glaucine	PAM	5-HT_2A_	[94]
Compound 58	NAM PAM	5-HT_2B_ 5-HT_2C_	[95]
VA012	PAM	5-HT_2C_	[96]
CTW0415	PAM	5-HT_2C_	[97]
CYD-1-79	PAM	5-HT_2C_	[98]
AP-267	PAM	5-HT_2C_	[99]
Compound 14	NAM	5-HT_2C_	[100]
